# Unplanned pregnancy and subsequent psychological distress in partnered women: a cross-sectional study of the role of relationship quality and wider social support

**DOI:** 10.1186/s12884-017-1223-x

**Published:** 2017-01-26

**Authors:** Katherine Barton, Maggie Redshaw, Maria A. Quigley, Claire Carson

**Affiliations:** 1 MSc student, Nuffield Department of Population Health, University of Oxford, Old Road Campus, Oxford, OX3 7LF UK; 2 National Perinatal Epidemiology Unit, Nuffield Department of Population Health, University of Oxford, Old Road Campus, Oxford, OX3 7LF UK

**Keywords:** Unintended pregnancy, Unplanned pregnancy, Psychological distress, Depression

## Abstract

**Background:**

Research into the impact of unintended pregnancy on the wellbeing of women tends to focus on pregnancies ending in either termination or lone motherhood. Unintended pregnancy is common in partnered women, but little is known about the association between unintended pregnancy and postpartum affective disorders, such as depression and anxiety in this group. Poor relationship quality and lack of social support are considered risk factors for psychological distress (PD). We examined the association between unplanned motherhood and subsequent PD in partnered women, for whom evidence is sparse, accounting for the role of relationship quality and social support.

**Methods:**

Data for 12,462 partnered mothers were drawn from the first survey of Millennium Cohort Study, completed at 9 months postpartum. Women reported whether their baby was planned, and how they felt when they discovered that they were pregnant. Pregnancy intention is categorised as “planned”, “unplanned/happy”, “unplanned/ambivalent” and “unplanned/unhappy”. PD was assessed using the modified 9-item Rutter Malaise Inventory. Social support was measured by a composite score for perceived support, and a measure of actual support from friends and family. Relationship quality was assessed using a modified Golombok-Rust Inventory of Marital State. The effect of pregnancy intention on the odds of PD at 9 months was estimated, adjusting for potential confounding factors. All analyses were weighted for response and design effects.

**Results:**

In total 32.8%(weighted) (4343/12462) of mothers reported an unplanned pregnancy: 23.3 wt% (3087) of mothers felt happy, 3.5 wt% (475) ambivalent, and 6.0 wt% (781) unhappy upon discovery. Unplanned pregnancy was associated with a significantly increased odds of PD compared to planned (OR 1.73 (95%CI: 1.53, 1.95)). This was more pronounced among women who reported negative or ambivalent feelings in early pregnancy (OR 2.72 (95%CI:2.17, 3.41) and 2.56 (95%CI:1.95, 3.34), respectively), than those who reported positive feelings (OR 1.39 (95%CI:1.21, 1.60)). Adjustment for relationship quality, in particular, reduced odds of PD after unplanned pregnancy (e.g. from 2.19 (95%C: 1.74, 2.74) to 1.63 (95% CI: 1.29, 2.07 in the unplanned, unhappy group compared to the planned).

**Conclusions:**

A third of partnered mothers reported that their pregnancy was unintended, yet this group is under-researched. Unplanned motherhood was associated with increased risk of PD at 9 months postpartum, particularly among women who felt unhappy or ambivalent at the start. The roles of relationship quality and social support require further investigation, as possible means to intervene and improve maternal wellbeing.

## Background

Definitions of pregnancy intention are used interchangeably and are often subjective. “Unplanned” is a widely applied term attributed to a variety of circumstances of pregnancy.[1] Unplanned pregnancies can be broken down by varying degrees of pregnancy intention (e.g. mistimed versus unwanted conceptions) or by the mother’s emotional reaction to the pregnancy (e.g. happy, ambivalent or unhappy).[2, 3] For this reason, newer measures of pregnancy intention attempt to capture the complex nature of this concept by treating planning status as a continuum.[1] Unplanned pregnancy is common worldwide; it is estimated that approximately 30 million births occurred globally after unplanned pregnancy in 2012.[4] Such pregnancies can result in adverse health outcomes for both mother and child [5, 6].

Although unplanned pregnancy is often associated single motherhood, prevalence is high among women who are cohabiting or married. The US National Survey of Family Growth found that in 2006–2010, approximately 23% of pregnancies occurring in marriage were unplanned, as were approximately 51% of those occurring amongst cohabiting mothers [2]. Similarly, the third National Survey of Sexual Attitudes and Lifestyles in Great Britain found that approximately 5% and 18% of married and cohabiting mothers respectively, described their pregnancy as unplanned [7], and a further 19% of married mothers and 34% of cohabiting mothers stated that they felt “ambivalent” about their pregnancy [7]. These results highlight that unplanned pregnancy occurs in a substantial number of married/cohabiting couples in the developed world.

At the population level in the UK, teen mothers constitute a small minority of all pregnancies [8], and a greater number of women in their late 20s and 30s become mothers after an unplanned pregnancy than do teenagers.[7] Despite this, research into the impact of unplanned pregnancy among partnered and non-teen mothers is sparse. Some researchers have examined the progression of unplanned pregnancies in mixed populations of both partnered and single mothers. [5, 6] As a result, short-term sequelae of such pregnancies, such as later presentation for prenatal care [6], are assumed to be common among both partnered and single mothers. However, the lack of research into partnered mothers specifically, means that the manner in which unplanned pregnancies progress in this population is not well understood. Given the impact that lone parenthood has on women’s wellbeing, any effect of unplanned parenthood in partnered women is likely to be masked in an analysis that combines single and partnered women. Postpartum mental health is an example of one such area in which research is lacking among partnered mothers. There is little evidence available as to if and how an unplanned pregnancy may affect the mental health of partnered women in the first year after giving birth.

Psychological distress (PD) can be defined as a state of emotional suffering characterised by symptoms of depression and anxiety,[9] and may encompass depression, anxiety and low mood. Maternal depression and anxiety are known to have negative consequences for the mother, family relationships,[10] and her child,[11] and are an important facet of postpartum health. It could be hypothesised that the disruption to life caused by an unplanned conception carried to live delivery may increase likelihood of PD. However, evidence for any such association remains equivocal, [12, 13] particularly in partnered women.

Maternal wellbeing may be influenced by her own characteristics (education, age, socioeconomic status) [14] as well as her experience of pregnancy and birth (such as emergency delivery, or preterm birth) [15]. In addition, adverse health behaviours such as smoking and alcohol are more prevalent among women experiencing an unplanned pregnancy [7], and are associated with anxiety and depressive symptoms.

Mothers reporting unplanned pregnancy may also experience poorer quality relationships with partners, and may receive lower levels of social support relative to those who planned their pregnancy.[5, 16] Higher levels of marital conflict and lower participation of the child’s father in childcare are reported, for example.[5] A survey of German mothers found those reporting the lowest levels of social support in the first trimester also had the lowest proportion of pregnancies described as planned at this time point.[16] Factors associated with poor relationship quality and low levels of social support have each been shown previously to be significantly associated with risk of depressive symptoms postpartum.[17–19] Furthermore, lack of support from family and friends has been shown to act as a link between stressful life events and depression/anxiety postpartum.[20] It could thus be hypothesised that poorer relationship quality and lower levels of social support will influence the relationship between unplanned pregnancy and PD.

Using data from the UK Millennium Cohort Study, this study examines the relationship between unplanned pregnancy in partnered mothers and PD at 9 months postpartum, taking into account the influence of key covariates including relationship quality and social support.

## Methods

### Millennium cohort study

The Millennium Cohort Study (MCS) is a nationally representative UK-based prospective cohort study tracking the lives of infants born to 18,552 families between 2000 and 2002. The first survey was conducted when the index child was 9 months old, on average. The MCS used Electoral Wards as the primary sampling unit and was stratified by country, and by level of deprivation. In England the wards were stratified by ethnic minority population (wards with >25% Black or Minority Ethnic group make up this stratum), by deprivation (poorest 25% wards by child poverty index make up the deprived stratum, while all other wards are considered advantaged stratum). In Scotland, Wales and Northern Ireland only deprivation was used for stratification. Within each of the selected wards, families or carers of a random sample of children from the child benefit register were contacted. [21, 22]. The response rate was 82%, and 99% of main respondents were the birth mother of the cohort baby. Interviews gathered data on circumstances of pregnancy and birth, sociodemographic characteristics of the family and parents’ psychological adjustment to the presence of a new child. Analyses were restricted to natural mothers of the cohort child who were married or cohabiting with a partner at the birth of their child and at 9 months postpartum, and who had complete data available on pregnancy planning, outcome and key covariates.

### Pregnancy intention

Pregnancy intention was assessed in two ways – all analyses were completed using maternal report of pregnancy planning (planned/unplanned) as the main exposure. Next mother’s stated pregnancy intention and her self-reported feelings when she found out she was pregnant were combined in order to place her into a category of intention status as follows: “Planned” (stated the pregnancy was planned); “unplanned, happy” (stated the pregnancy was a surprise and recalled being happy/very happy upon discovery of pregnancy); “unplanned, ambivalent” (stated the pregnancy was unplanned and they “were not bothered either way”) and “unplanned, unhappy” (stated the pregnancy was a surprise and recalled being unhappy/very unhappy upon discovery of pregnancy).

### Measures of psychological distress, relationship quality and social support

Maternal psychological distress (PD) was assessed using the 9-item modified version of the Rutter Malaise Inventory [23, 24], where a score of ≥4 indicated the participant was exhibiting symptoms of PD. Items include questions such as: “Do you often feel miserable and depressed?”, “Do you often get into a violent rage?”, “Do you often get worried about things?”, and were answered yes or no. The scale has been used in a number of cohort studies, and was developed to achieve a reliability (Cronbach’s alpha) of at least 0.70 [24].

Relationship quality was assessed using a modified 7 item version of the Golombok Rust Inventory of Marital State (GRIMS) score [24, 25], which includes questions on different aspects of the partnership (e.g. “my husband/partner doesn’t listen to me”, “my husband/partner is usually sensitive to and aware of my needs”, “we can always make up quickly after an argument”) assessed on a 5-point Likert scale (strongly agree to strongly disagree) and summed to produce an overall score (Cronbach’s alpha 0.85). Those who responded “can’t say” or “don’t know” to any item were excluded.

Perceived social support was assessed using several questions in the survey, where mothers were asked to indicate on a 5 point likert scale if they agreed or disagreed with the following statements: “There are other parents I can talk to about my experiences”, “I have no one to share my feelings with” (emotional support) and “If I had financial problems, I know my family would help if they could” (practical support availability). Social contact (as a proxy for support) was evidenced in (‘Sees friends or family once a week or more’, answered ‘yes/no’) and was analysed as a binary variable.

### Statistical analyses

The characteristics of the different pregnancy intention groups were described and compared using chi-squared tests. Multivariable logistic regression was used to estimate the odds of PD at 9 months postpartum in mothers reporting unplanned pregnancies relative to planned pregnancies, controlling for potential confounders. Variables were adjusted for in several stages due to the large number of potential confounders, as follows:

1. **Sociodemographic Variables**: Maternal age (years), Education level (highest level of academic or vocational education, equivalent to National Vocational Qualifications: degree or higher (NVQ 4/5), school qualifications at 18 (NVQ 3), school qualifications at end of compulsory schooling (NVQ1/2), none or overseas qualifications only), ethnicity (Black and Minority Ethnic group/not BME), household socioeconomic status (highest National Statistics Socioeconomic Classification, according to current or last known job of woman and her partner, and grouped as Professional/Managerial, Intermediate, Routine/Manual, long-term unemployed and never-worked).
2. **Pregnancy**-**Related Variables**: Firstborn child, smoked during pregnancy (none/gave up/kept smoking), gestation at which pregnancy was confirmed (weeks), preterm birth (<37weeks), delivery method (normal vaginal/instrumental/planned c-section/emergency c-section), multiple birth.
3. **Relationship quality** (measured as described above).
4. **Social support** (measured as described above).

At each stage, variables were included in the final model if they were associated with the outcome (p < 0.05), after adjustment for all other variables in that model. The final model comprised those that were significant in each of the 4 separate models. All analyses were completed using STATA 14.0 [19], first examining the effect of pregnancy planning (planned/unplanned) and then of intention (planned/happy/ambivalent/unhappy). Geographical clustering and disproportionate stratification of the sample included in MCS permitted adequate representation of all four UK countries, disadvantaged areas and areas of England with a higher population of ethnic minorities [21] (20). Descriptive and logistic regression analyses used weights to account for the stratified clustered nature of the data and non-response [22] (21). Reported percentages means, odd ratios and confidence intervals are weighted, and frequencies are unweighted.

## Results

### Characteristics of partnered mothers reporting unintended pregnancy

12,462 partnered women were included in the analysis; the characteristics of the study population are shown in Table 1. 33% of mothers reported their pregnancy as unplanned (23% reported a positive response to the pregnancy, 6% reported a negative response, and 4% reported ambivalence). Compared to the planned pregnancies, mothers reporting unplanned pregnancy tended to be younger, of lower SES and had fewer qualifications. A smaller proportion of women in the unplanned group were first time mothers, and a higher proportion smoked prior to pregnancy and did not give up cigarettes while pregnant. Women reporting an unplanned pregnancy were also less likely to be married and, on average, had a lower relationship quality score and poorer support, than the mothers reporting a planned pregnancy.

**Table 1:** Characteristics of partnered mothers, according to pregnancy intention. True counts and weighted proportions and means are shown

	All	Planned pregnancy	Unplanned Pregnancy			
			All	Happy	Ambiv.	Unhappy
N	12,462	8,119	4,343	3087	475	781
(weighted %)	(100)	(67.2)	(32.8)	(23.3)	(3.5)	(6.0)
Sociodemographic characteristics						
Maternal age (yrs), mean (SD)	30.5 (5.3)	31.1 (4.7)	29.3 (6.0)***	29.2 (6.0)***	28.9 (6.3)***	30.0 (6.0)***
Ethnicity (BME)	8.6	7.3	11.2***	10.9****	14.1****	10.6**
Socioeconomic status			****	****	****	****
Managerial/professional	54.3	60.2	42.3	43.7	33.2	42.3
Intermediate	20.9	20.1	22.6	22.7	25.4	20.9
Routine/Manual	22.9	18.1	32.5	31.3	38.6	33.7
Long-term unempl/never	1.9	1.6	2.5	2.3	2.8	3.1
Education			****	****	****	****
High (NVQ 4 + 5)	39.3	43.8	30.0	31.6	22.5	28.2
Medium (NVQ 3)	15.0	14.7	15.7	16.4	14.7	13.8
Low (NVQ1 + 2)	36.5	34.5	40.5	38.6	45.9	44.8
Other/none of the above	9.2	7.0	13.8	13.4	17.0	13.2
Pregnancy-related factors						
First child	40.3	41.2	38.5**	43.7	28.8****	24.1****
Gestation at confirmation of pregnancy, mean (SD)	7.1 (3.2)	6.7 (2.7)	7.8 (3.9)****	7.8 (3.7)****	8.7 (4.7)****	8.3 (4.2)****
Preterm birth	7.1	6.6	8.1**	7.9*	8.3	8.8
Multiple birth	1.6	1.8	1.2*	1.1*	1.3	1.5
Type of delivery			****	*	****	****
Normal vaginal	66.0	64.3	69.6	67.3	76.5	74.3
Instrumental	11.1	12.1	9.1	10.0	8.5	5.9
Planned section	10.0	10.4	9.1	9.4	5.9	10.1
Emergency section	12.8	13.2	12.2	13.3	9.1	9.8
Smoking in pregnancy			****	****	****	****
None	71.4	76.4	61.1	63.1	54.6	57.3
Gave up	12.6	11.1	15.5	15.8	14.9	14.8
Kept smoking	16.0	12.4	23.4	21.1	30.6	28.0
Partnership characteristics						
Relationship quality (mean GRIMS, SD): higher is better	28.0 (4.6)	28.4 (4.4)	27.3 (4.8)***	27.7 (4.7)***	26.5 (4.9) ***	26.0 (5.2) ***
Marital status (% married)	72.9	80.4	57.7****	58.8***	49.2***	58.2***
Social support						
“No-one to share my feelings with”, % agree	5.3	4.5	6.9	5.6	10.2	10.0
“I have other parents I can talk to”, % disagree	9.3	8.1	11.6	10.4	14.5	14.2
“My family would help, if they could”, % disagree	5.9	5.8	6.1	5.0	4.8	11.2
Limited social contact - Sees friends or family < weekly	8.7	8.4	9.5	8.8	9.6	12.1**
Psychological Distress, modified Malaise Inventory score ≥4						
Yes	11.6	9.6	15.6****	13.0****	21.4****	22.4****

*P < 0.05; **P < 0.01; ***P < 0.001; ****P < 0.0001 for chi-squared test, where compared to the Planned pregnancy group

When the unplanned pregnancy group were divided by their response to finding out about their pregnancy, there were distinct differences between the groups. A greater proportion of those who reported that they were happy when they found out they were expecting a baby were first time mothers (43.7%, compared to 28.8% in ambivalent and 24.1% in unhappy groups). They were also more likely to be in the highest SES group, and to have higher qualifications, than the women who reported feeling ambivalent or unhappy (although not as high as the planned group, see Table 1). The mothers in the ambivalent group were the youngest, on average, and had the lowest SES and educational attainment. This group also had the highest proportion of BME women, and the lowest proportion of married women. Among the women who reported that their pregnancy was unplanned, more of those who reported feeling unhappy were pregnant with a second or subsequent child.

The unplanned, ambivalent and unplanned, unhappy groups were more likely to report poor social support, and more limited social contact than the planned group. The ambivalent and unhappy groups also had lower relationship quality scores at 9 months post-partum.

### Pregnancy intention and psychological distress at 9 months postpartum

The prevalence of psychological distress (PD) at 9 months was 11.6% overall. The prevalence was lowest in the mothers reporting a planned pregnancy (9.6%), and increased in the unplanned groups, from 13.0% in those who reported a positive reaction when they found out that they were pregnant, to 21.4% in those who reported ambivalent feelings, and 22.4% in those who reported negative feelings.

The unadjusted analysis suggests that compared to the planned group, women who experienced an unplanned pregnancy were 1.73 (95%CI: 1.53, 1.95) times more likely to exhibit signs of PD at 9 months post-partum (see Table 2). The association was most pronounced among those who had a negative response when they found out they were pregnant (OR 2.72 (95%CI: 2.17, 3.41)). After adjusting for confounding of socioeconomic, demographic and pregnancy-related factors, the association was reduced but remained significant in all groups. Odds of PD at 9 months for those women reporting unplanned pregnancies were 1.47 (1.30, 1.66) times those of women who described their pregnancy as planned. The odds of PD were highest in those reporting being unhappy about their pregnancy, at 2.19 (95%CI: 1.74, 2.74).

**Table 2:** The association between pregnancy intention and subsequent psychological distress in partnered women, OR (95% Confidence Interval)

	Pregnancy Intention groups, compared to ‘Planned’							
	Unplanned Pregnancy							
Model	All			Happy	Ambivalent	Unhappy		
	OR (95% CI)	F	p	OR (95% CI)	OR (95% CI)	OR (95% CI)	F	p
Unadjusted	1.73 (1.53,1.95)	78.97	<0.0001	1.39 (1.21,1.6)	2.56 (1.95,3.34)	2.72 (2.17,3.41)	39.50	<0.0001
1: + Socio-demographic	1.55 (1.37,1.76)	49.60	<0.0001	1.26 (1.09,1.45)	2.19 (1.66,2.88)	2.46 (1.96,3.07)	29.21	<0.0001
2: 1 + Preg-Related	1.47 (1.30,1.66)	36.89	<0.0001	1.22 (1.06,1.41)	1.96 (1.48,2.59)	2.19 (1.74,2.74)	21.08	<0.0001
3: 2 + Rel. Quality	1.29 (1.13,1.47)	14.12	0.0002	1.13 (0.97,1.32)	1.65 (1.22,2.25)	1.63 (1.29,2.07)	8.12	<0.0001
4: 3 + Social Support	1.26 (1.10,1.45)	10.98	0.0010	1.13 (0.97,1.32)	1.47 (1.07,2.02)	1.59 (1.25,2.02)	5.99	0.0005

Design degrees of freedom (df) for models: 389 (unplanned), 387 (happy, ambiv, unhappy)

F = F statistic, from Wald test

p = p value from Wald test

Model 1: ethnicity, household socio-economic status

Model 2: model 1, plus firstborn child, smoking in pregnancy

Model 3: model 2, plus GRIMS (relationship quality)

Model 4: model 3, plus emotional and instrumental support

### The role of relationship quality and wider social support

Adjustment for relationship quality and perceived social support reduced the OR further. The association between pregnancy intention and PD was no longer statistically significant in the unplanned, happy group (OR_adj_ 1.13 (95%CI: 0.97, 1.32), but remained significant in the ambivalent and unhappy groups, who were around 1.5 times more likely to experience PD at 9 months than the planned group, even after adjusting for the effect of relationship quality and social support.

## Discussion

Previous research has reported prevalence of unplanned pregnancy in new mothers in the UK to be 41% [26]. Here we find that even when restricting to partnered mothers, approximately 1 in 3 report their pregnancy as unplanned (23% reported positive feelings about the unplanned pregnancy, 4% ambivalent feeling and 6% had a negative response). Consistent with previous research, mothers reporting unplanned pregnancies tended to be in more disadvantaged groups and to report lower levels of relationship quality and social support. In many cases, these effects were particularly pronounced in those reporting ambivalent or negative feelings about their unplanned pregnancy.

There was a clear increased risk of psychological distress (PD) at 9 months postpartum after unplanned pregnancy that remained after adjustment for confounders, and the effect was more marked for the mothers who reported feeling ambivalent or negative when they realised that they were pregnant, than for those who felt happy or very happy at that time. Analysis of the roles of relationship quality and levels of wider social support suggest that these factors play an important role in the association between pregnancy intention and later symptoms of psychological distress.

### Pregnancy intention and psychological distress

Unplanned pregnancy among partnered mothers was associated with an increased risk of PD at 9 months postpartum, relative to a planned pregnancy: OR 1.22, 1.96 and 2.19 for “unplanned/positive”, “unplanned/ambivalent” and “unplanned/negative” groups, respectively, after adjustment for sociodemographic, economic and pregnancy-related factors. This is consistent with the existing literature [12, 14]. The stress associated with the transition into parenthood [14] may be exacerbated in cases of unplanned pregnancy by factors relating to socioeconomic position, such as the increased financial pressures of a new child, and psychological readiness for motherhood. Little research exists to examine the impact of unplanned pregnancy on the lives of partnered mothers, specifically. Single motherhood is strongly associated with reporting a pregnancy as unintended or mistimed [7], and so in studies that include both single and partnered women the substantial impact of lone parenting on women’s wellbeing can mask more subtle effects. Findings of the present study demonstrate that a strong association remains between unplanned pregnancy and PD, even after the effects of single motherhood are removed.

### Relationship quality

Among partnered mothers, factors such as postpartum marital closeness [17], partner support and occurrence of interpersonal violence [19] are significantly associated with risk of depressive symptoms in the postpartum period, demonstrating the importance of a good quality relationship to the mental health of partnered mothers. Adjustment for relationship quality reduced the odds of PD, suggesting that this plays a significant role in the association between unplanned pregnancy and development of PD. As well as practical assistance, a stable relationship may increase resilience and aid the mother’s development of coping mechanisms in the transition to an unplanned parenthood. Unplanned pregnancy may have a detrimental impact on the quality of the partner relationship, increasing the risk of psychological distress in this group.

### Social support

Research into the roles of perceived and actual support after unplanned pregnancy is limited. In this study population, women who described their pregnancy as unplanned reported lower levels of perceived support and less frequent contact with friends and family, particularly in the case of those reporting ambivalent or negative feelings around their unplanned pregnancy. Adjustment for perceived support reduced the odds of PD after unplanned pregnancy, further emphasising the potential role it plays in the association between the unplanned pregnancy and PD. Perceived support is well-established as a major predictive factor for postpartum affective disorders, but literature on actual support is less consistent [19]. However, its buffering role has been demonstrated in a range of high-stress contexts [10]. Such support may decrease feelings of isolation and reduce the impact of stressful events on an individual’s life [27].

### Strengths and limitations

Few studies to date have examined the impact of unplanned pregnancy on subsequent wellbeing in partnered mothers, a group who represent a significant proportion of all unplanned pregnancies. Use of the MCS population provides a large sample of women who had a baby after an unplanned pregnancy, and permits generalisation of findings to partnered mothers across the UK. Key potential confounding factors were included in the analysis.

A number of limitations must be considered. Retrospective ascertainment of pregnancy intention may be affected by post-hoc rationalization [28]. However, it has been reported that collecting data on pregnancy intention in this way does not affect estimates of either number or consequences of unintended births [29]. We analysed women who reported feeling happy about their unplanned pregnancy separately from those who said that they felt negative or ambivalent when they realised that they were pregnant, to explore the impact that underlying desire for a child may have on PD after an unplanned conception. However, this classification was based on asking mothers to recall their emotional response which may be affected by current mood or depressive symptoms. To mitigate against the potential impact of current mood, we also analysed all ‘unplanned’ pregnancies together, which did not take feelings about the pregnancy into account – and the significant association with PD was evident. While the data used here are approximately 15 years old, unplanned pregnancy remains a common occurrence in the UK. Cultural changes around single parenthood may (perhaps) have influenced the impact of an unplanned pregnancy for single women since the data were collected, but it is difficult to see what societal changes will have altered the psychological impact unintended motherhood among partnered women. We therefore believe these results continue to be generalizable to partnered women.

MCS used a slightly modified version of the GRIMS questions to assess relationship quality, offering an option of “can’t say” as a response which was not part of the original instrument design. The <1.5% of women who responded in this way were coded to ‘missing’ for the individual components of GRIMS; 6% of women were excluded because they had one or more items missing for the GRIMS. If this response is associated with negative outcomes, then we may have underestimated the prevalence of poor relationship quality. This would serve to underestimate any observed effect between pregnancy intention and PD.

The data analysed here are cross-sectional and therefore represent a snapshot of the study participants’ lives when their babies were 9 months old, on average. As a consequence, these results apply to women’s wellbeing at around 9 month postpartum and associations between pregnancy intention and PD at different times may vary. Past history of psychiatric illness is a risk factor for postpartum depression [10]. Depending on the timing of previous episodes, a prior history of psychiatric disorders might affect the intention to get pregnant, or consistency of contraceptive use [30], well as social support and relationship quality during pregnancy and after delivery. The majority of couples who were married or cohabiting at the birth of their child remained in these groups at 9 months, there were insufficient numbers to assess the impact of changing relationship status (e.g. cohabiting to married). Data were not available to allow us to assess the quality of partner relationship or mental health prior to or during pregnancy, so we could not investigate this further.

Relationship quality and social support were treated and interpreted here as confounding factors. However, this could be a simplification of the complex inter-relationships between psychological wellbeing, relationship quality, social support and pregnancy intention. It could be postulated that relationship quality and social support in fact act as mediating or moderating factors in this relationship, given that each has been previously shown to be associated with reporting a pregnancy as unplanned and with PD postpartum [5, 16–19]. The exact roles of relationship quality and social support in this association thus require further research in order to disentangle these complex relationships and determine their specific pathways at work.

## Conclusion

The present study found that partnered mothers reporting unplanned pregnancies represent a large proportion of all births and are at a significantly increased risk of PD at 9 months postpartum. This group also tend to report poorer relationship quality and lower levels of social support, both of which appear to drive the observed association but do not fully explain it. The effect is more marked among women who reported a negative or ambivalent response to finding out they were pregnant. Further research into the role of relationship quality and social support is needed to confirm their role in the development of PD in this group. Women who experience an unplanned pregnancy, even in a stable partnership, may benefit from more post-natal follow-up, particularly if they have limited support.

## Abbreviations

BME: Black or Minority Ethnic group
GRIMS: Golombok Rust Inventory of Marital State
MCS: Millennium Cohort Study
NVQ: National Vocational Qualification
OR: Odds ratio
PD: Psychological Distress
SES: Socioeconomic Status
wt%: Weighted percentage
